# Supplementary figures and images for: Domain coupling in allosteric regulation of SthK measured using time-resolved transition metal ion FRET
Source: eLife. 2025 Aug 12;14:RP106892. doi: 10.7554/eLife.106892 (PMC12342821; doi:10.7554/eLife.106892)

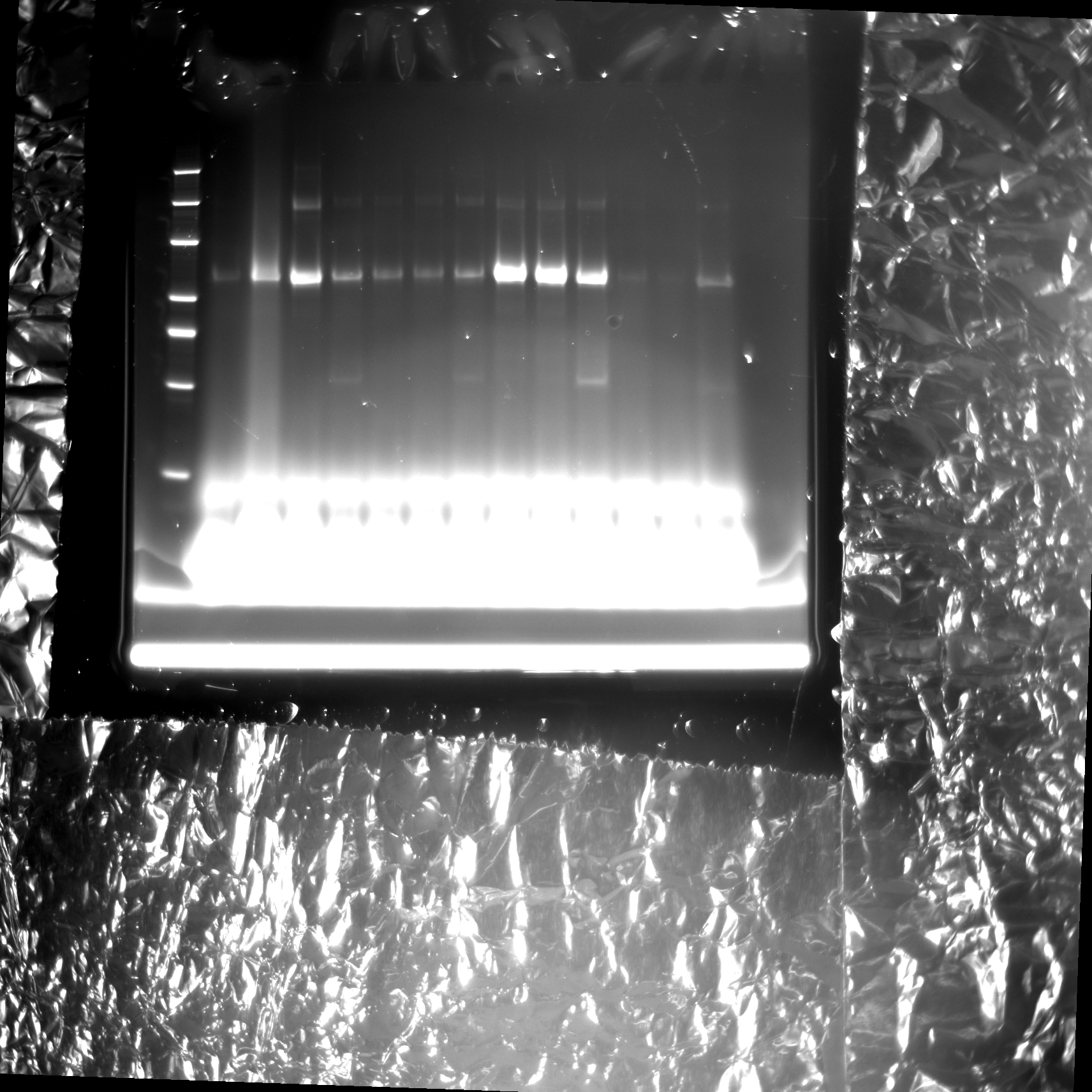

Supplement: Figure 5—source data 1. [file elife-106892-fig5-data1.zip › Figure 5-source data 1-InGel-Original.jpg]

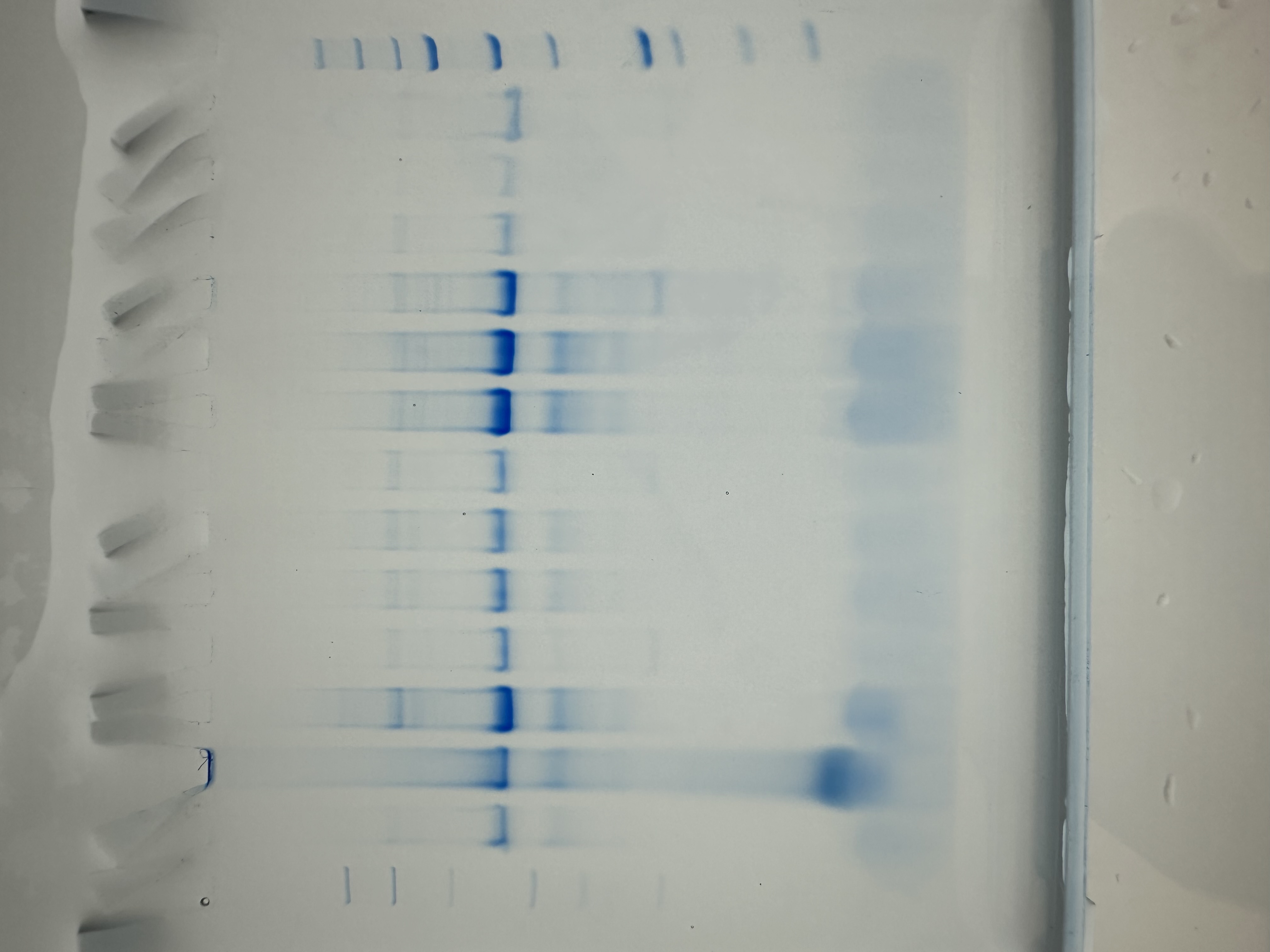

Supplement: Figure 5—source data 1. [file elife-106892-fig5-data1.zip › Figure 5-source data 1-Coomassie-original.jpg]

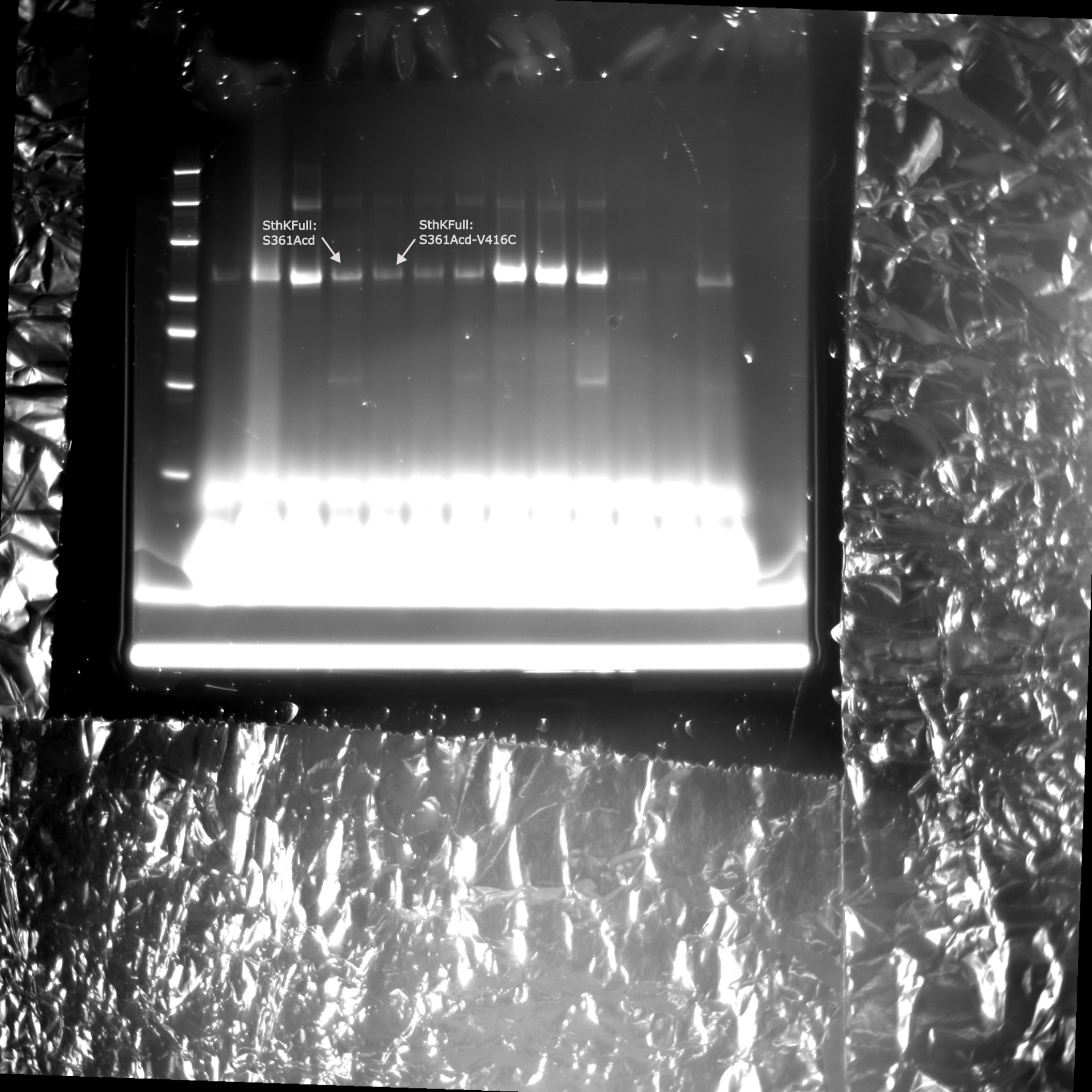

Supplement: Figure 5—source data 2. [file elife-106892-fig5-data2.zip › Figure 5-source data 2-InGel-Labeled.jpg]

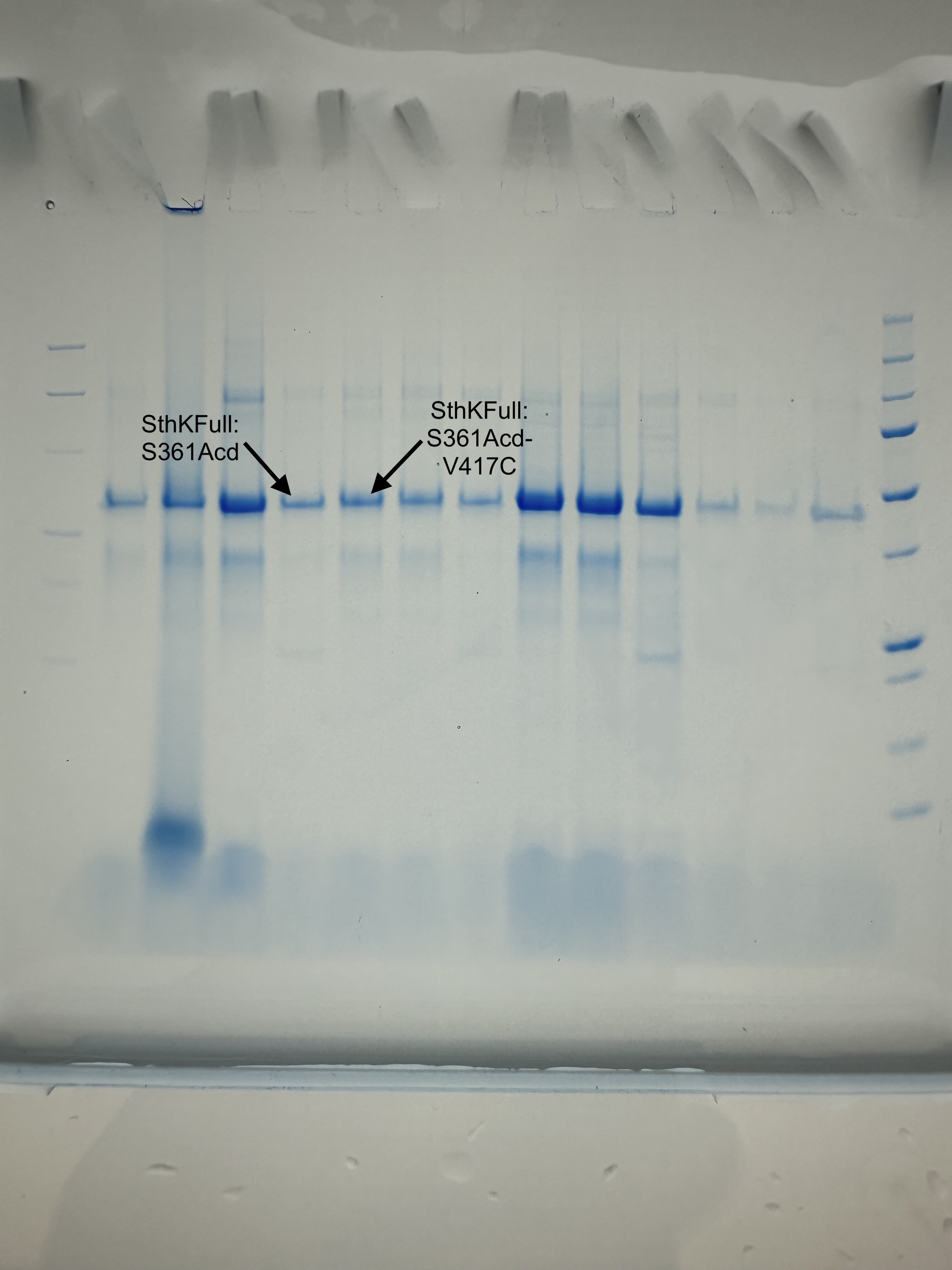

Supplement: Figure 5—source data 2. [file elife-106892-fig5-data2.zip › Figure 5-source data 2-Coomassie-labeled.jpg]
